# Intelligent Detection Method of Gearbox Based on Adaptive Hierarchical Clustering and Subset

**DOI:** 10.1155/2022/6464516

**Published:** 2022-08-30

**Authors:** Huimiao Yuan, Yongwei Tang, Huijuan Hao, Yuanyuan Zhao, Yu Zhang, Yu Chen

**Affiliations:** ^1^Qilu University of Technology (Shandong Academy of Sciences), Shandong Computer Science Center (National Supercomputer Center in Jinan), Shandong Key Laboratory of Computer Networks, Jinan 250014, China; ^2^School of Mechanical Engineering, Shandong University, Key Laboratory of High Efficiency and Clean Mechanical Manufacture, Jinan 250100, China

## Abstract

Deep learning uses mechanical time-frequency signals to train deep neural networks, which realizes automatic feature extraction and intelligent diagnosis of fault features and gets rid of the dependence on a large number of signal processing technology and experience. Aiming at the problem of misclassification of similar samples, a fault diagnosis algorithm based on adaptive hierarchical clustering and subset (AHC-SFD) is proposed to extract features and applied to gearbox fault diagnosis. Firstly, the adaptive hierarchical clustering algorithm is used to analyze the characteristics of different data, and then the data set is clustered into multiple feature groups; finally, according to the feature group, the SubCNN model is established for multiscale feature extraction, so as to carry out fault diagnosis. The test results show that the fault recognition rate achieved by the proposed method is more than 99.7% on the gearbox dataset, and the method has better generalization ability.

## 1. Introduction

Major accidents caused by mechanical equipment failure [1] constantly alert people to ensure the safe and reliable operation of equipment, especially the mechanical equipment failure at the key core of the production line will bring significant shutdown losses to the whole production line, not only causing huge economic losses, but also endangering personal safety in serious cases. The online monitoring, fault diagnosis, and prediction of mechanical equipment [2, 3] play an important role in improving equipment operation reliability, optimizing operation and maintenance strategies, and are crucial to the maintenance of mechanical equipment. Traditional intelligent fault diagnosis methods need to master a large number of signal processing techniques to extract relatively accurate feature parameters. At the same time, if the shallow model is used to characterize the relationship between signal and fault, and the diagnosis ability and generalization ability are insufficient, it is difficult to meet the actual needs of fault diagnosis under big data.

In recent years, the application of deep learning in fault diagnosis of complex industrial systems has begun to take shape [4]. Lei et al. [5, 6] proposed a big data health monitoring method based on denoising self-encoder (DAE) for mechanical equipment, which has realized a variety of fault diagnosis for planetary gears, reflecting the powerful ability of deep learning to extract mechanical vibration signal characteristics. Yu and Zhao [7–9] effectively integrated DAE and EN to solve the problem of noise interference in fault diagnosis, effectively detect abnormal samples in industrial processes, and isolate fault variables from normal variables. Nguyen et al. [10–12] proposed a deep learning network composed of automatic encoder and softmax classifier to identify bearing faults of different degrees. DBN is more combined with other technologies to solve the problem of fault diagnosis. Since CNN was used to identify bearing faults in 2016, fault diagnosis performance and scope of application have been continuously improved. Hoang and Kang [13–16] proposed a new method based on CNN for rolling bearing fault diagnosis. By using the effectiveness of CNN in image classification, the CWRU bearing data set can achieve 100% diagnosis accuracy. Based on resnet-50, a transfer learning convolution neural network TCNN is proposed by Wen et al. [17, 18] for fault diagnosis, and the prediction accuracy is significantly better than other DL models and traditional diagnosis methods. The application of RNN in fault diagnosis began to recover in 2015. Abed et al. [19, 20] used RNN for bearing fault diagnosis and realized accurate detection and classification of bearing faults under nonstationary conditions. Pan et al. [21–23] proposed a method for bearing fault classification by combining one-dimensional CNN and LSTM, and the experimental test accuracy is 99.6%.

Although the above algorithm has been applied in mechanical equipment fault diagnosis, there is still a lot of room to improve the fault recognition rate. Feature extraction is a key part of fault diagnosis. It is found that for samples with similar features and belonging to different patterns, a single model will extract similar features, resulting in false recognition [24] and a reduction in the accuracy of fault diagnosis. In view of the above problems, referring to the idea of subset [25, 26], this study proposes a multiscale feature extraction fault diagnosis algorithm model AHC-SFD based on adaptive hierarchical clustering and applied to gearbox fault diagnosis. The test results show that the proposed method can achieve the fault recognition rate achieved by the proposed method is more than 99.7% on the gearbox dataset and has better generalization ability.

## 2. Gear Fault Diagnosis Algorithm Based on Adaptive Hierarchical Clustering and Subset

Gear boxes generally work in the environment with strong noise and complex structure, and the collected vibration signals are easily affected by external factors. To fully develop the feature extraction ability of the CNN network, this study proposes a fault diagnosis algorithm based on adaptive hierarchical clustering and subset. First, all data obtained the optimal clustering results through adaptive hierarchical clustering, and a multiscale feature extraction module is designed according to the clustering results to realize the classification of fault data.

### 2.1. Adaptive Hierarchical Clustering

**Figure 1:**
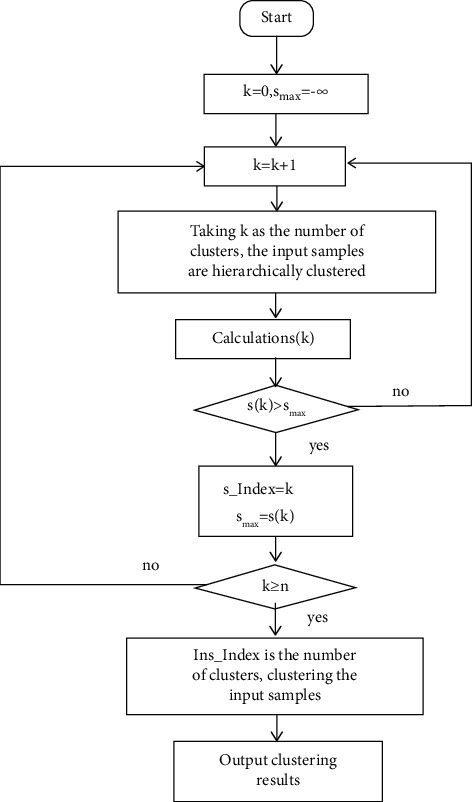
Adaptive hierarchical clustering flow chart.

The number of clusters is an important parameter that affects the clustering effect, but before clustering, it is often necessary to set the number of clusters to take a fixed value. As the amount of data changes, the original parameter values cannot optimize the clustering result of the algorithm. Combined with the characteristics of vibration signals, an adaptive hierarchical clustering (DIANA) algorithm is proposed in this study. The clustering contour coefficient is used as the index of clustering effectiveness evaluation, so that it can adaptively determine the number of clusters according to the value of self-defined discriminant function. The process is shown in Figure 1.

The specific algorithm flow chart is as follows:

(1) Extract the average value of each original vibration signal to form a feature sample set *X*={*x*_1_, *x*_2_,…, *x*_num_} , *U*={*u*_1_, *u*_2_,…, *u*_*C*_} indicates fault type set
(2) Start clustering, make *k*=0, *s*_max_=−*∞*;
(3) Let *k*=*k*+1, take *k* as the number of clusters, and perform hierarchical clustering on the input training samples (DIANA);
(4) Calculate the contour coefficient *s*(*k*),
  (1) $$\begin{array}{c} a\left(i\right)=\frac{1}{n_{c}-1}\sum_{j\in C_{c},i\neq j}d\left(i,j\right). \end{array}$$
In equation (1), *n*_*c*_ represents the number of samples of class *c*, *C*_*c*_ represents the samples of class *c*, and *d*(*i*, *j*) represents the absolute distance between samples *i* and *j*;
  (2) $$\begin{array}{c} b\left(i\right)=\begin{array}{c} \min\\ p,p\neq c \end{array}\left[\frac{1}{n_{p}}\sum_{j\in C_{p},i\in C_{c}}d\left(i,j\right)\right]. \end{array}$$
In equation (2), *p* denotes a mark other than Class *c*, *n*_*p*_ represents the number of samples not of class *c*, *C*_*p*_ represents a sample that is not class *c*, *C*_*c*_ is the sample of class *c*, and *d*(*i*, *j*) is the absolute distance between samples *i* and *j*;
  (3) $$\begin{array}{c} s\left(i\right)=\frac{b\left(i\right)-a\left(i\right)}{\max\left[a\left(i\right),b\left(i\right)\right]}. \end{array}$$
In equation (3), *a*(*i*) represents the average distance between sample *i* and all other samples belonging to the same type of fault, and *b*(*i*) represents the minimum value of the average distance between sample *i* and all samples in each class of nonclass *i* fault;
  (4) $$\begin{array}{c} s\left(k\right)=\frac{1}{\text{num}}\sum_{i=1}^{\text{num}}s_{i}. \end{array}$$
In equation (4), *s*_*i*_ is the contour coefficient of the sample individual, num is the number of samples in the feature sample set, and *k* is the number of clusters;
(5) When *s*(*k*) > *s*_max_, then *s*_Index=*k* and *s*_max_=*s*(*k*), perform step 7;
(6) When *s*(*k*) ≤ *s*_max_, return to step 3;
(7) Judge whether *k* is less than *n*, where *n* indicates the number of dataset types:
When *k* ≥ *n*, *s*_Index is the number of clusters and the clustering results are output;
When *k* < *n*, repeat step 3.

### 2.2. Multiscale (Subset) Feature Extraction

**Figure 2:**
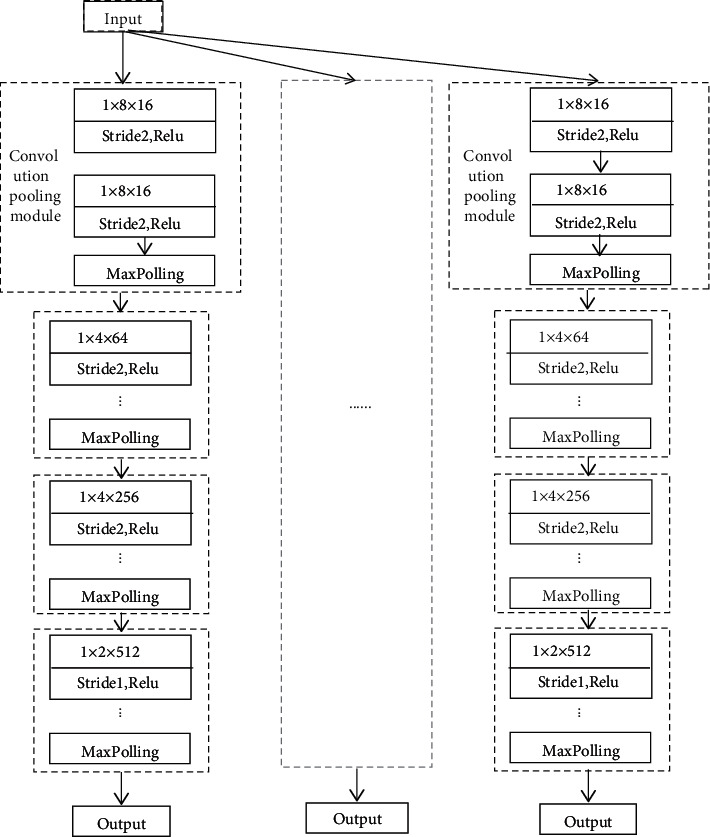
Multiscale feature extraction module.

In order to maximize the extraction of feature information from training data and quickly realize iteration, this study designs a multilayer and multichannel multiscale feature extraction module based on the CNN. The structure is shown in Figure 2. The branch structure of each subset (12 layers in total) is the same, in which the convolution kernel sizes of the 8-layer convolution layers are 1*∗*8, 1*∗*8, 1*∗*4, 1*∗*4, 1*∗*4, 1*∗*2, and 1*∗*2, the number of channels is set to 16, 16, 64, 64, 256, 256, 512, and 512, and the step size is set to 2, 2, 2, 2, 2, 1, and 1. The relu activation function is used behind each convolution layer, and the max pool layer of 4 adopts the 1*∗*2 structure. Finally, the extracted feature information is output.

### 2.3. AHC-SFD Diagnostic Algorithm

**Figure 3:**
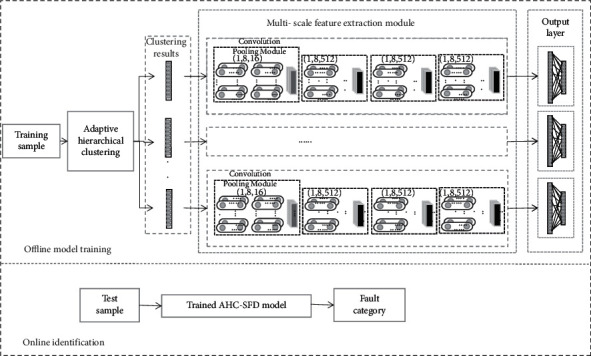
Flow chart of adaptive hierarchical clustering and subset fault diagnosis.

The flow chart of adaptive hierarchical clustering and subset fault diagnosis proposed in this study is shown in Figure 3. The mean value of each vibration signal is used as the input of adaptive hierarchical clustering to obtain the optimal clustering results. The labeled samples corresponding to the results are input to the multiscale feature extraction module to obtain more effective fault data features. Finally, the features extracted by the multifeature extraction module are transformed into one-dimensional data through the fully connected layer. Output the fault diagnosis result through softmax function.

## 3. Experimental Verification and Analysis

**Figure 4:**
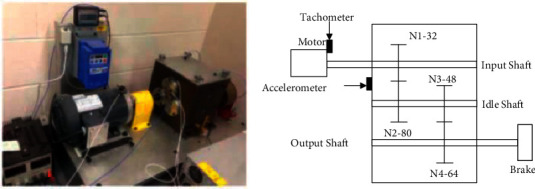
Gearbox experimental system.

In order to evaluate the effectiveness and accuracy of fault diagnosis of the AHC-SFD network model, the gearbox dataset is used for experimental verification. The data are collected from a reference two-stage gearbox, the gear speed is controlled by a motor, and the torque is provided by a magnetic brake, which can be adjusted by changing its input voltage. A 32-tooth pinion and an 80-tooth pinion are installed on the first stage input shaft, the second stage consists of a 48-tooth pinion and a 64-tooth pinion. Input shaft speed is measured by tachometer, and gear vibration signal is measured by accelerometer, as shown in Figure 4.

### 3.1. Fault Dataset Description and Processing

**Figure 5:**
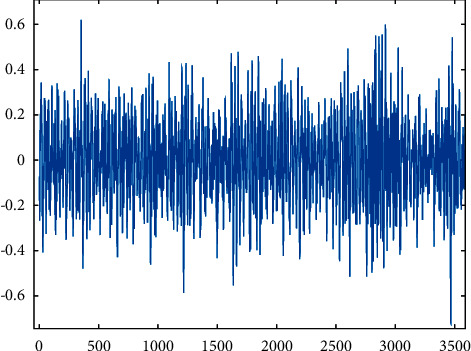
Health.

**Figure 6:**
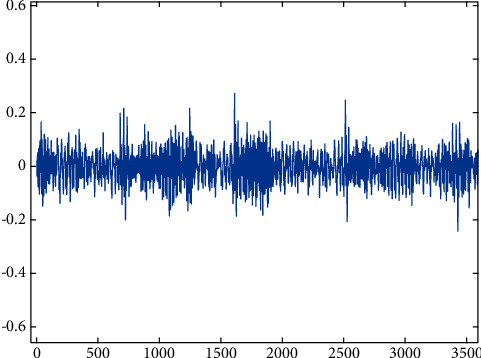
Missing teeth.

**Figure 7:**
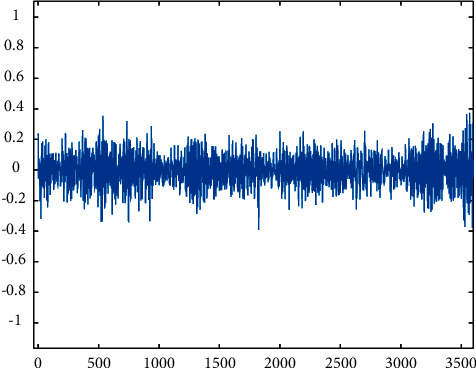
Root cracking.

**Figure 8:**
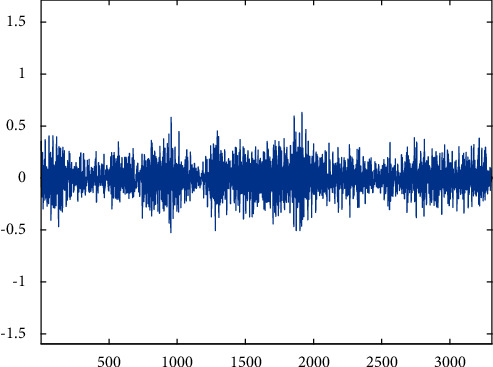
Peeling.

**Figure 9:**
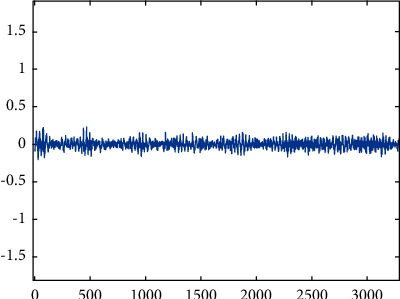
Tip cutting5.

**Figure 10:**
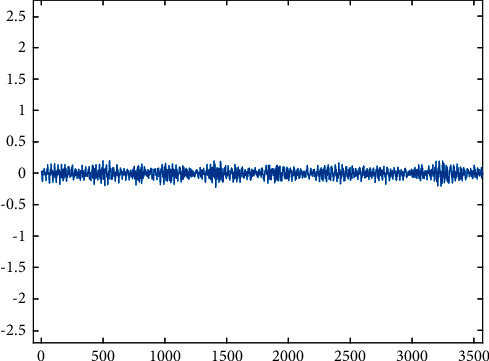
Tip cutting4.

**Figure 11:**
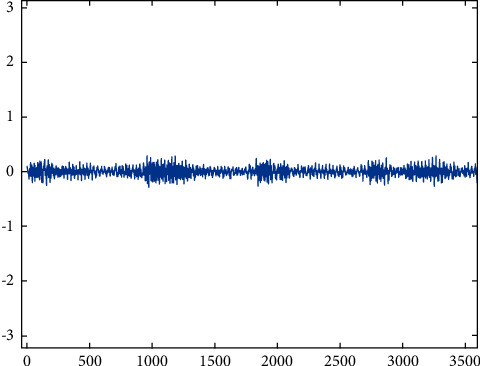
Tip cutting3.

**Figure 12:**
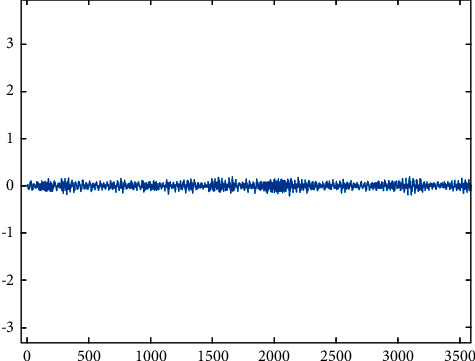
Tip cutting2.

**Figure 13:**
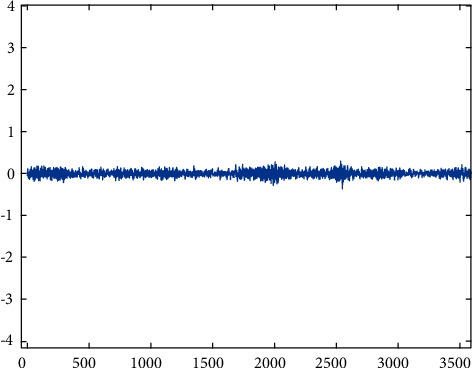
Tip cutting1.

The pinion on the input shaft introduces 9 different gear conditions, including five different severity labels, such as health, missing teeth, root cracking, peeling, and tip cutting. The number of samples in each status tag is the same. The collected data are roughly divided into training samples and test samples in the proportion of 4 : 1. Each sampling sample is set to 3600 points. The dataset is described in Figures 5–13 and Table 1.

### 3.2. Adaptive Hierarchical Clustering

#### 3.2.1. Refactoring Input Data Format

The dataset collected by the test-bed is a one-dimensional vibration signal sequence. In order to reduce the clustering time and carry out the adaptive hierarchical clustering operation quickly and effectively, this study takes the one-dimensional vibration signal with 3600 sampling points as the average value and takes the average value as the input value of the adaptive hierarchical clustering. The specific operation is as follows:

(5) $$\begin{array}{c} X=\frac{\sum_{i=1}^{3600}x_{i}}{3600}. \end{array}$$

In equation (5), *x*_*i*_ represents the *i*-th eigen value of a sample and *X* represents the average value of a sample.

#### 3.2.2. Result Output

**Figure 14:**
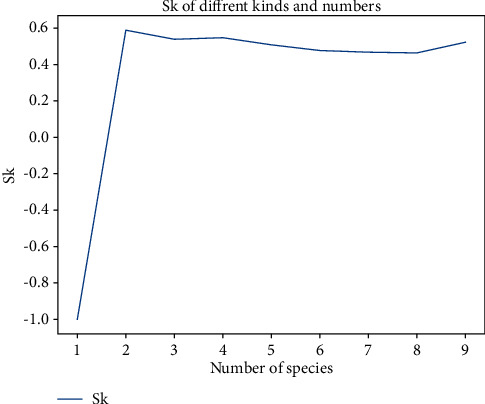
Cluster contour coefficients of different cluster numbers.

The principle of adaptive clustering is to obtain a certain clustering result, so that the distance between classes is as large as possible, the distance within a class is as small as possible, and the classes have good separability. It can be seen from 2.1 that the cluster contour coefficient is used as the index for cluster effectiveness evaluation in this study. The closer the cluster contour coefficient is to 1, the better the clustering result is. The closer it is to −1, the worse the clustering result is. In this study, the number of clusters is set between [1, 9]. During clustering, the cluster contour coefficients obtained with the change of the number of clusters is shown in Figure 14. It can be clearly seen that when the number of clusters are 2, the cluster contour coefficient (Sk) is the largest. Therefore, the branch of the multiscale feature extraction module is set to 2.

### 3.3. Improved CNN Network

#### 3.3.1. Grouping Label Data According to Clustering Results

Use labeled data; the labeled data samples are (*x*^(1)^, *y*^(1)^), (*x*^(2)^, *y*^(2)^),…, (*x*^(*m*)^, *y*^*m*^), *x*^(*i*)^ represents the feature vector, and *y*^(*i*)^ ∈ {1,2,…, *t*} represents the fault type. According to the clustering results in 3.2.2, the label data (one-dimensional vibration signal) is divided into two groups. The two groups are divided into training samples and test samples according to the ratio of 39 : 11 and 19 : 6, respectively. The description of the training and testing datasets is shown in Table 2.

#### 3.3.2. Data Standardization Operation

In order to better speed up the network model training, make the data easy to calculate and obtain more generalized results, the input data are standardized, and the vibration signal data are mapped to the (0,1) interval by using the normalization equation. The mathematical expression is as follows:

(6) $$\begin{array}{c} z_{i}=\frac{x_{i}-\begin{array}{c} \min\\ 1\leq f\leq F \end{array}\left\{x_{f}\right\}}{\begin{array}{c} \max\\ 1\leq f\leq F \end{array}\left\{x_{f}\right\}-\begin{array}{c} \min\\ 1\leq f\leq F \end{array}\left\{x_{f}\right\}}. \end{array}$$

In equation (6), *z*_*i*_ represents the preprocessed data, *x*_*i*_represents the frequency value of the vibration signal, $\begin{array}{c} \text{min}\\ 1\leq f\leq F \end{array}\left\{x_{f}\right\}$ and $\begin{array}{c} \text{max}\\ 1\leq f\leq F \end{array}\left\{x_{f}\right\}$ represent the minimum and maximum values of frequency in each group of vibration signals, and *f* represents the number of each vibration signal.

#### 3.3.3. Diagnostic Result Output

In order to evaluate the difference between the normalized prediction result and the corresponding sample label, the cross entropy function is used to calculate the error loss value. The mathematical expression is as follows:

(7) $$\begin{array}{c} J\left(\theta \right)=-\frac{1}{m}\sum_{i=1}^{m}\sum_{r=1}^{t}\left(I\left\{y^{\left(i\right)}=r\right\}\times \;\log\frac{e^{x_{i}^{t}}\Delta c_{k}}{\sum_{k=1}^{m}e^{x_{i}^{t}}\Delta c_{k}}\right). \end{array}$$

In equation (7), *J*(*θ*) represents the loss function, *I*{Δ} represents the logical indication function (when the value is true, *I* = 1, otherwise *I* = 0), and *y*^(*i*)^ represents the *i*-th real label of the fault.

The weight matrix *θ* is iteratively updated by means of gradient descent. The iterative equation is as follows:

(8) $$\begin{array}{c} \theta_{j}=\theta_{j}-\alpha \frac{\partial J\left(\theta \right)}{\partial \theta_{j}}. \end{array}$$

In equation (8), *θ*_*j*_ represents the weight matrix of the *j*-th update.

#### 3.3.4. Model Parameter Structure

The experiment was implemented on a Linux computer using Pycharm platform, Python as the programming language, and PyTorch deep learning framework.

During network training based on stochastic gradient descent, the multilayer back-propagation of the error signal can easily lead to “gradient dispersion” (too small gradient will make the returned training error signal extremely weak) or “gradient explosion” (too large gradient will lead to Nan in the model). With the increase of network depth, training becomes more and more difficult. Considering the network lightweight, during the experiment, the Adam optimizer is used to continuously update the network training parameters. The batch size is set to 30 and the number of iterations is 200. This study introduces the early stopping mechanism. By monitoring the changing value of the training set loss function between adjacent iterations during the training process, early stopping can terminate the model training in time to prevent the model from overfitting. The learning rate is 0.0005. The model is built on the basis of convolutional neural network model, so the parameter design is similar to the convolutional neural network, and the parameter design is shown in Table 3.

### 3.4. Result Analysis

**Figure 15:**
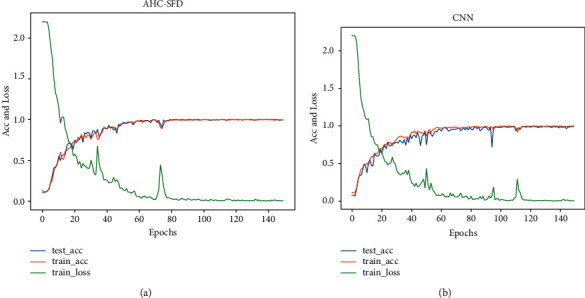
AHC-SFD and CNN experimental results.

To verify whether the method has a high diagnostic rate and good generalization ability, the experimental results in this study are compared with those using only the CNN. The experimental results are shown in Figure 15.

**Figure 16:**
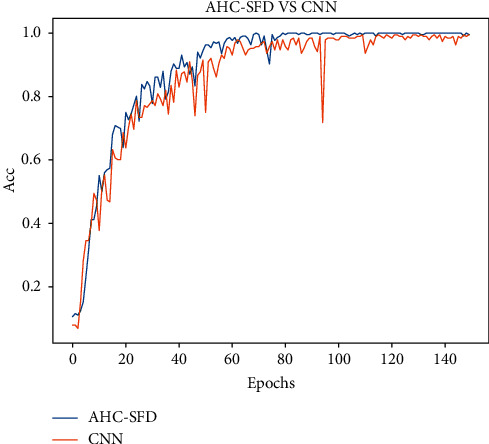
AHC-SFD Vs CNN.

The comparison results of AHC-SFD and CNN on the test set are shown in Figure 16.

It can be seen from the comparison results in Figures 15 and 16 that after 140 epochs, the accuracy of AHC-SFD algorithm on the test set reaches 99.7%, while the accuracy of the CNN algorithm on the test set is only 98.9%. Therefore, the diagnostic methods in this study tend to be faster, more stable, with higher accuracy and stronger generalization ability.

**Figure 17:**
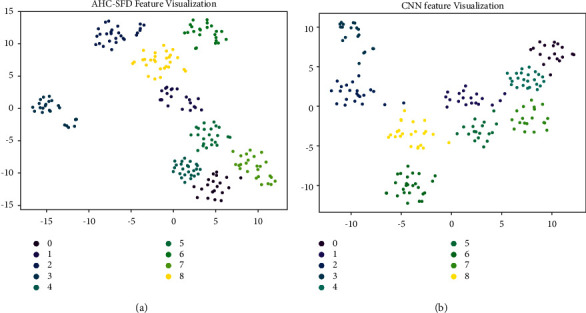
AHC-SFD and CNN feature visualization.

In order to further demonstrate the learning ability of the model for different categories of features, the t-SNE dimension reduction algorithm in flow pattern learning is introduced to visualize the features learned by the full connected layer. The experimental results are shown in Figure 17.

It can be seen from the scatter plot Figure 17 that the method AHC-SFD in this study has identification errors in the samples of class 0 and class 7, and the other samples are gathered at the corresponding positions. However, CNN features have recognition errors in class 1, class 2, class 5, and class 8 samples, and there are many overlaps in class 1 and class 5 samples. It can be seen that AHC-SFD has stronger feature learning ability than the CNN.

## 4. Conclusion

The AHC-SFD algorithm established in this study is a diagnosis algorithm based on adaptive hierarchical clustering and subset, which has the following three advantages: (1) the AHC-SFD algorithm directly takes the original vibration signal as the input of 1D-CNN, which can obtain the characteristics of vibration signal to the greatest extent. (2) A grouping method based on adaptive hierarchical clustering is proposed, which analyzes the characteristics of different data and then clusters the dataset into multiple feature groups. (3) A multiscale feature extraction module is proposed to reduce the misclassification of similar samples, thus ensuring the maximum extraction of effective information into the data. It is verified on the gearbox dataset that the diagnostic accuracy is better than the single-channel CNN model.

## Figures and Tables

**Table 1:** Gearbox dataset.

Fault information		Sample information		Category information
Fault type	Fault degree	Sample length	Number of samples	Category tag
Health	0	3600	104	0
Missing tooth	0	3600	104	1
Root crack	0	3600	104	2
Spalling	0	3600	104	3

Tip cutting	5 (lightest)	3600	104	4
	4	3600	104	5
	3	3600	104	6
	2	3600	104	7
	1 (most serious)	3600	104	8

**Table 2:** Training test dataset.

Grouping information	The number of training samples	The number of test samples
Group I	360	102
Group II	360	104

**Table 3:** Parameter design.

The number of layers	Structure name	Structural parameters	The number of channels	Output size
	Input	(1,3600)	1	(1,3600)
1	Convolution layer	(1,8,2)	16	(1,1797)
2	Convolution layer	(1,8,2)	16	(1,895)
3	Pool layer	(1,2)		(1,447)
4	Convolution layer	(1,4,2)	64	(1,222)
5	Convolution layer	(1,4,2)	64	(1,110)
6	Pool layer	(1,2)		(1,55)
7	Convolution layer	(1,4,2)	256	(1,26)
8	Convolution layer	(1,4,2)	256	(1,12)
9	Pool layer	(1,2)		(1,6)
10	Convolution layer	(1,2,1)	512	(1,5)
11	Convolution layer	(1,2,1)	512	(1,4)
12	Pool layer	(1,2)		(1,2)
13	Full connection layer	(1024)		(1024)
14	Full connection layer	(50)		(50)
15	Output layer	(9)		(9)

## Data Availability

The data set used in this article can be obtained from the corresponding author upon request.
